# Selection of DNA Aptamers against Glioblastoma Cells with High Affinity and Specificity

**DOI:** 10.1371/journal.pone.0042731

**Published:** 2012-10-02

**Authors:** Dezhi Kang, Jiangjie Wang, Weiyun Zhang, Yanling Song, Xilan Li, Yuan Zou, Mingtao Zhu, Zhi Zhu, Fuyong Chen, Chaoyong James Yang

**Affiliations:** 1 Department of Neurosurgery, The First Affiliated Hospital of Fujian Medical University, Fuzhou, Fujian, China; 2 State Key Laboratory of Physical Chemistry of Solid Surfaces, Key Laboratory for Chemical Biology of Fujian Province, Key Laboratory of Analytical Science and Department of Chemical Biology, College of Chemistry and Chemical Engineering, Xiamen University, Xiamen, Fujian, China; Univ of Bradford, United Kingdom

## Abstract

**Background:**

Glioblastoma is the most common and most lethal form of brain tumor in human. Unfortunately, there is still no effective therapy to this fatal disease and the median survival is generally less than one year from the time of diagnosis. Discovery of ligands that can bind specifically to this type of tumor cells will be of great significance to develop early molecular imaging, targeted delivery and guided surgery methods to battle this type of brain tumor.

**Methodology/Principal Findings:**

We discovered two target-specific aptamers named GBM128 and GBM131 against cultured human glioblastoma cell line U118-MG after 30 rounds selection by a method called cell-based Systematic Evolution of Ligands by EXponential enrichment (cell-SELEX). These two aptamers have high affinity and specificity against target glioblastoma cells. They neither recognize normal astraglial cells, nor do they recognize other normal and cancer cell lines tested. Clinical tissues were also tested and the results showed that these two aptamers can bind to different clinical glioma tissues but not normal brain tissues. More importantly, binding affinity and selectivity of these two aptamers were retained in complicated biological environment.

**Conclusion/Significance:**

The selected aptamers could be used to identify specific glioblastoma biomarkers. Methods of molecular imaging, targeted drug delivery, ligand guided surgery can be further developed based on these ligands for early detection, targeted therapy, and guided surgery of glioblastoma leading to effective treatment of glioblastoma.

## Introduction

Glioblastoma is the most common and highest-grade primary malignant brain tumor in adults, with over 10,000 death each year in the US alone [1]. Being one of the most aggressive cancers, Glioblastoma is characterized by rapid growth rate and highly invasive capacity to infiltrate to critical neurological areas within the brain. Most standard clinical treatments fail to treat glioblastoma [2], [3], [4] because of its notoriously resistance to apoptosis. Over the decades, in spite of advances in surgical techniques, radiotherapy and chemotherapy, no effective therapeutic approaches are available [2]. Currently, patients with glioblastoma are usually treated with surgical excision, followed by external beam radiotherapy and/or chemotherapy. However, the median survival of this disease is generally less than one year and most patients succumb to the disease within two years after diagnosis [5], [6]. Mainly due to the infiltrative ability of the glioblastoma cells and highly heterogeneous environment of brain tissue, without any guidance the complete removal of the tumor is almost impossible and thus the recurrence rate is high. The detailed molecular characterization of glioblastoma can not only accurately define the molecular pathology of tumor region thus guiding the surgery, but also lay the foundation for rationally designed, targeted therapies. Therefore, it is of great significance to discover such ligands that can characterize glioblastoma at the molecular level, which would be beneficial for early detection, diagnosis, targeted therapy and guided surgery of glioblastoma to improve the therapeutic efficacy and survival rate for this disease.

Aptamers are highly structured single-stranded oligonucleotides that can bind to a variety of target molecules with high affinity and specificity, including small molecules such as ATP, large biomolecules such as proteins, and even whole cells or bacteria [7], [8], [9]. Compared with traditional antibodies, aptamers have many advantages including ease of synthesis and modification, stability at room temperature, lack of immunogenicity, and rapid tissue penetration [10], [11], [12], [13]. Because of these advantages, aptamers show great application prospects in fundamental research, drug selection, clinical diagnosis and therapy [14], [15], [16]. For example, mucagen, the first aptamer-based drug towards age-related macular degeneration (AMD), is available on market, and some other aptamers such as aptamer AS1411, which is specific for nucleolin, are in clinical trials now [17].

Aptamers are generated by a method named Systematic Evolution of Ligands by EXponential enrichment (SELEX) [18], [19]. SELEX process is to discover those oligonucleotides from a large oligonucleotide library that can fold into unique 3D structures to interact with a specific target with high specificity and affinity [20]. Cell-SELEX was specifically developed to select aptamers against live cells to obtain cell-specific binding ligands. Thus, Cell-SELEX is a particularly promising selection strategy for various applications, including cancer diagnostics and therapy. Specifically, by adopting Cell-SELEX, useful probes can be developed to potentially differentiate tumor cells from normal cells, as well as to differentiate two different types of cancers [20]. One major advantage of Cell-SELEX over conventional SELEX is that it can be performed even without identification of target proteins. Aptamers can be generated against diseased or differentiated cells without prior knowledge of the surface marker [14]. Cell-SELEX can discover aptamers for unknown biomolecules, which have the potential to be elucidated as disease biomarker [21], [22], [23]. Moreover, because aptamers are developed for biomolecules on the cell surface that are in their native state and would therefore represent their natural folding structures as well as their distribution, the aptamers will bind to the real folded conformation of intact proteins. Additionally, since the selection has been done with whole cells, a panel of aptamer probes can be generated against many receptor proteins on the cell membrane surface. As a result, it is possible to profile the molecular characteristics of the target cancer type [24]. Many cancer cell lines, including CEM, Ramos, and A172, have been used as the target for aptamer selection [25], [26], [27]. A variety of methods for cancer cell detection, molecular imaging, and targeted therapy have been successfully developed based on cell-specific aptamers, demonstrating the potential of aptamers for cancer diagnostics and treatment [28], [29], [30].

In order to identify specific aptamers against human glioblastoma for biomarker discovery, cancer recognition, diagnosis and targeted therapy, human glioblastoma cell line U118-MG as target cell and human astroglial cell line SVGp12 as control cell were employed for cell-SELEX, two glioblastoma-specific aptamers named GBM128 and GBM131 were selected and validated. These two aptamers neither recognize normal astraglial cells, nor do they recognize other normal and cancer cell lines tested. Clinical tissues were also examined and the results showed that these two aptamers can bind to different clinical glioma tissues but not normal brain tissues, demonstrating the binding affinity and selectivity of these two aptamers were retained in complicated biological environment. Overall, our results confirmed the high binding affinity and specificity of our selected aptamers, which have the potential applications in glioblastoma studies, such as early cancer diagnosis, and targeted therapy.

## Results and Discussion

**Figure 1:**
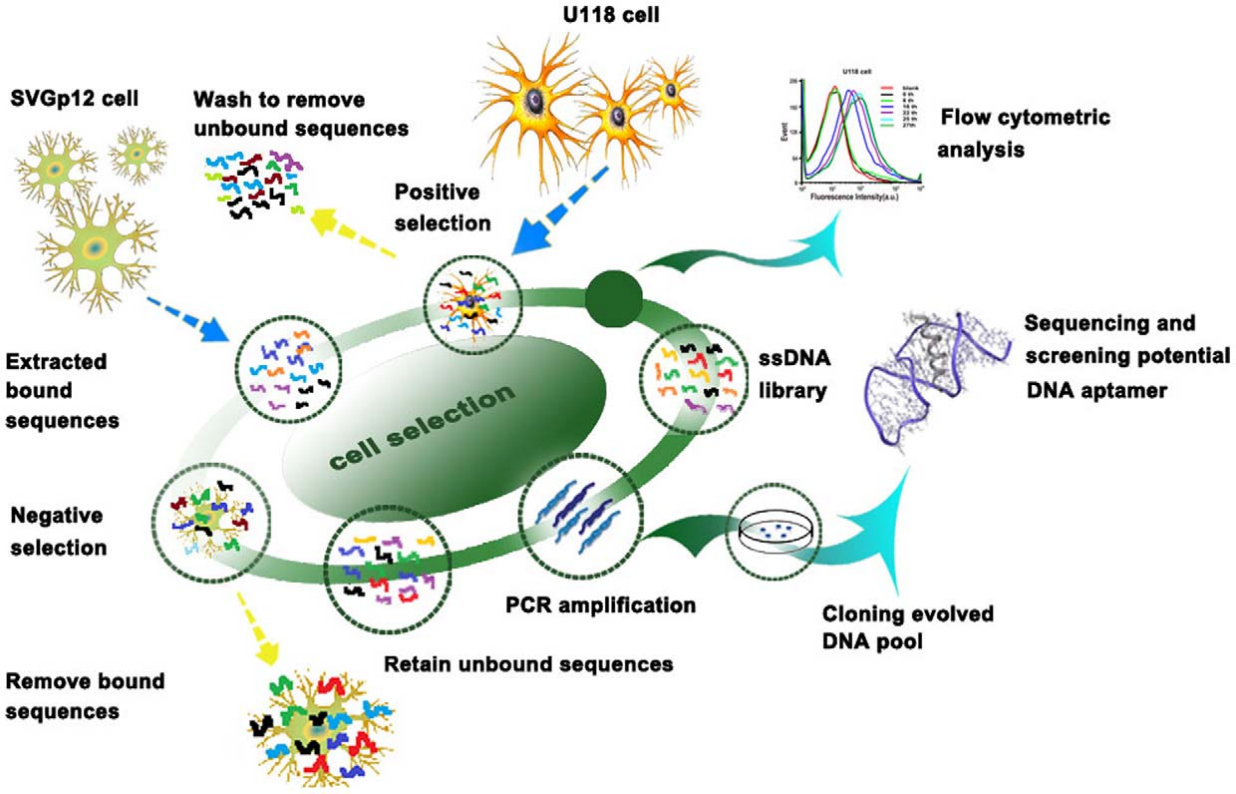
Schematics of systematic evolution of DNA aptamers against U118-MG cells.

**Figure 2:**
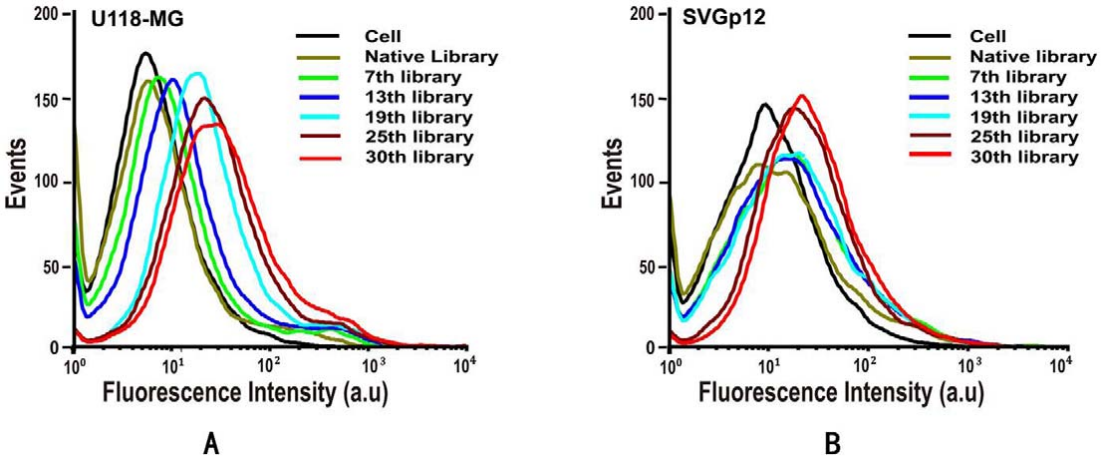
The binding affinity assay of the enriched pools with U118 MG and SVGp12 cells. (A) The fluorescence intensity of U118-MG cells binding with selected pools increased gradually as the selection progressed, indicating that the enhanced binding affinity of enriched pools. (B)The selected pools also showed increased affinity with SVGp12 cells, but the response was apparently weaker than with U118-MG cells. Final concentration of FAM-labeled ssDNA pools was 250 nM in binding buffer.

In this report, U118-MG was chosen as the target cell line and SVGp12 as the negative cell line, both of which are adherent cell lines, and the selection was performed in the culture dish directly as an accurate representation of the native state of the membrane proteins. The cell-SELEX process in the present study started with the initial ssDNA pool followed by an iterative process to specifically amplify sequences with high binding affinity and specificity to U118-MG cells. Schematic illustration of DNA aptamer selection for U118-MG cells is shown in Figure 1. The SELEX procedure was performed according to the reference with minor modification [14], [20]. In short, the ssDNA library was first incubated with target U118-MG cells, the supernatant containing the ssDNA which didn't bind with U118-MG cells was then discarded. The adherent cells were washed several times and then scraped off from the bottom of the culture dish. Unlike the trypsin detachment that can destroy the cell surface proteins, the scraping method we applied to detach cells would not affect the binding of aptamers on cell membrane surfaces. The bound DNA was eluted by heating the ssDNA-cells complex at 95°C for 5 min in 500 μL of binding buffer. The eluted DNA was then incubated with SVGp12 cells (control cells) for counter selection on ice. Negative selection can remove sequences that bind to both negative and positive cell lines thus ensuring the enriched sequences recognize only target cells. After incubation, the supernatant was collected and desalted with a NAP-5 column and then amplified by PCR with biotin-labeled antisense primer. Through streptavidin-biotin reaction and alkaline denaturation, the selected sense ssDNA was separated from the biotinylated antisense ssDNA strand and used as the DNA library for next round of enrichment. During the selection, flow cytometric analysis was performed to monitor the progress of the selection process. With increasing numbers of selection rounds, the DNA probes with better binding affinity to the target cells were enriched. A large increase of fluorescence intensity was observed for U118-MG cells (target cells) as shown in Figure 2A, while the increase of fluorescence intensity for the control cell line was much smaller (Figure 2B), suggesting the successful enrichment of DNA probes that bind only to target U118-MG cells, not the control SVGp12 cells.

After 30 rounds of selection, the enriched DNA library was cloned and sequenced. DNA sequences from 246 clones were obtained, and grouped into 14 families according to their homology of random sequences and the secondary structures. Out of the 14 families, 12 sequences were chemically synthesized with FAM or Cy5 labeled at their 5′-end. Flow cytometric analysis was carried out to validate the binding affinity and specificity of these sequences. The results in Table 1 showed that the binding affinities of 9 aptamers out of 12 were found to be in the tens of nM range, confirming their high binding capability against target cell U118-MG. The specificity of these 9 aptamers was further tested by analyzing the binding of FAM-labeled aptamers to different glioblastoma cell lines and many other normal and cancer cell lines. As shown in Table S1, all the aptamers can bind to the target U118-MG cells, aptamer GBM10, GBM11, GBM17 and GBM34 can not only bind to target U118-MG cells and other glioblastoma cells but also control astroglial cells and all the other cell lines tested. This indicated that these 4 aptamers have high binding affinity with target cells but poor specificity. In contrast, aptamers GBM128 and GBM131 can only bind to target cancer cell line U118-MG and another glioblastoma cell line U87-MG but not with any other cell lines tested (Table S1). Figure S1 shows typical binding plots of GBM128 and GBM131 against target and control cell lines, indicating their excellent specificity. As a result, these two aptamer sequences were further characterized.

**Table 1:** Aptamer sequences and their dissociation constants (K_d_).

Name	Sequence	Percentage of total sequences(%)	K_d_ (nM)
GBM1	GAA TTC AGT CGG ACA GCG AGT TGT TGT TAG GGT GTT GGG TTG GGG TTT TAG GGT CCC TGT CGT CGA TGG ACG AAT ATC GTC TCC C	10.6	38±17
GBM7	GAA TTC AGT CGG ACA GCG GCA TTT ACA TTC CAT GGG AAA TTT CTC AAG CGG TAT TTC ATT TCG ATG GAC GAA TAT CGT CTC CC	8.1	46±11
GBM10	GAA TTC CGT CGG ACA GCG GTA TGC ATG GGA GGT TCT GGA GGG GGT GGG TTG GCG TGC GTT CCA GAT GGA CGA ATA TCG TCT CCC	7.3	29±7
GBM11	GAA TTC AGT CGG ACA GCG CGG TGT TGG TGG TGG GGT GGT TTG GGA ATC TGT TCG GCT GGA GAT GGA CGA ATA TCG TCT CCC	3	52±18
GBM17	GAA TTC AGT CGG ACA GCG GTG GTG CTT GTG TAT GGG GGT GGT TGG TGG GTT TTA TGC TGC TGG ATG GAC GAA TAT CGT CTC CC	1.2	27±13
GBM24	GAA TTC AGT CGG ACA GCG AGG AAA ATT TCG TTA TTT TCG TTT CCG GGA ACT CGG GCA TTT AGA GAT GGA CGA ATA TCG TCT CCC	3.7	31±14
GBM34	GAA TTC AGT CGG ACA GCG CGG TGT TGG TGG CGG GGG TGG TTG GGG GAT TTG GCT GTC CTG AGT GAT GGA CGA ATA TCG TCT CCC	5.3	19±8
GBM128	GAA TTC AGT CGG ACA GCG ACG GTG GGA GCC CCA AAT AAT TCT TGC GAT TAT TAG TGT AAG CGG ATG GAC GAA TAT CGT CTC CC	1.6	20±10
GBM131	GAA TTC AGT CGG ACA GCG GCA CTT GCG ACA CGT TTG TCG GGT AAA TGC GTG TAT TTT CTT TTC GAT GGA CGA ATA TCG TCT CCC	2	37±13

To reduce the endocytosis of the cells, the selection was performed by incubating the cells with the DNA library on ice, and initial binding affinity results were also obtained in the same way. To confirm their binding ability at 37°C which is physiological temperature, the target U118-MG cells were incubated with aptamers GBM128 and GBM131 at 4°C and 37°C for 30 min. As shown in Figure S2, both aptamers can bind to target U118-MG cells very well (Figure S2 A and B) at 4°C, but aptamer GBM128 lost its binding affinity to U118-MG cells at 37°C (Figure S2C). Aptamer GBM131 showed reduced, but still significant binding against U118-MG cells at 37°C (Figure S2D). Thus, aptamer GBM128 can only be used at 4°C and GBM131 can work at both 4°C and 37°C. The binding capability of aptamers GBM131 and GBM128 in complicated environment was further investigated. Taken cell growth medium as a model, target cells (5×10^5^) were incubated with 25 pmol FAM-labeled aptamers in 100 μL growth medium on ice for 30 min prior to flow cytometric analysis. The results in Figure S3 indicated that these two aptamers still bound with target U118-MG cells in cell growth medium.

**Figure 3:**
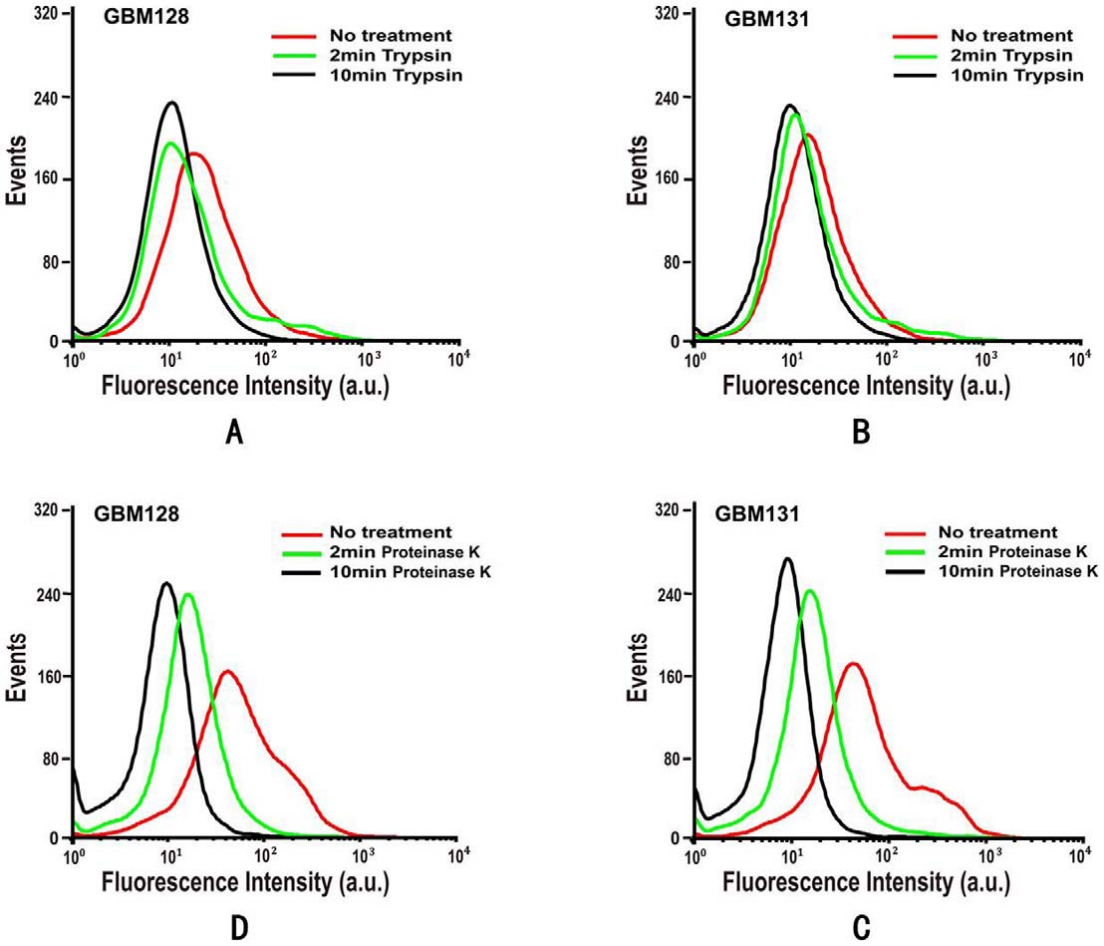
Binding affinity of aptamers GBM128 and GBM131 to trypsin or proteinase K treated U118-MG cells. After 2 min and 10 min trypsin or proteinase K treatment, the binding of GBM128 and GBM131 was apparently affected by trypsin digestion but totally quenched by proteinase K. The final concentration of FAM-labeled aptamers was 250 nM.

In order to determine whether the target of the aptamers is a membrane protein on the cell surface, FAM-labeled aptamers were incubated with trypsin or proteinase K treated cells on ice for 30 min and then analyzed by flow cytometry. As shown in Figure 3, all aptamers lost their binding abilities to their target cells treated with proteinase K. We also found that trypsin treatment was not as effective as that of proteinase K in decreasing aptamer's binding affinity, indicating that the binding entities of these two aptamers might resist to trypsin cleavage slightly. Overall, the decrease in binding efficiency after treatment with trypsin or proteinase K strongly implied that the aptamer targets on target cells were most probably membrane proteins.

**Figure 4:**
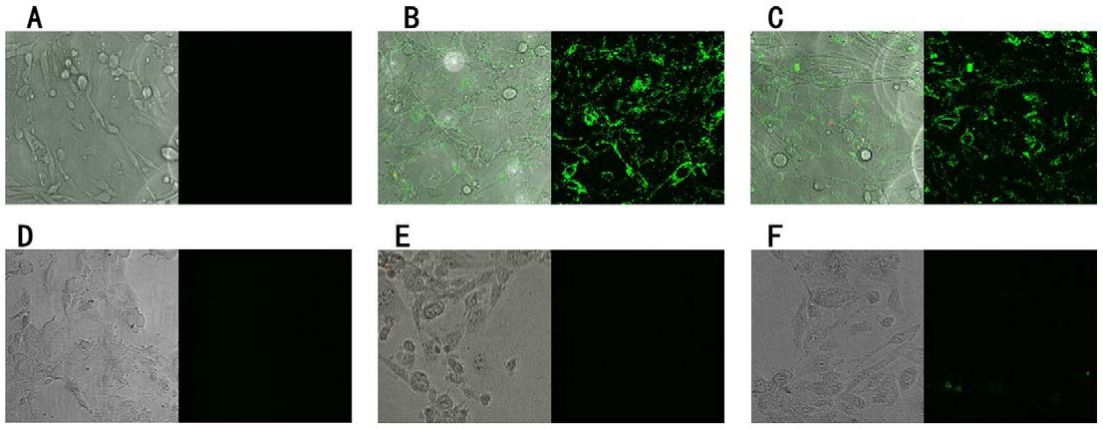
Confocal images of cultured U118-MG and SVGp12 cells staining with FAM-labeled aptamers. Cells were incubated with FAM labeled initial library and aptamers. The initial library showed the background binding and aptamers showed significant binding over the background. For U118-MG cells: A =  initial library; B =  GBM128; C =  GBM131. For SVGp12 cells; D =  initial library; E =  GBM128; F =  GBM131. The final concentration of FAM-labeled aptamers was 250 nM.

Immunohistological imaging and fluorescence microscopy are very useful tools for studying tumors in clinics. We further assessed whether these two aptamers could be used for glioblastoma imaging. The binding assays were performed in culture dishes directly. Cells were incubated with FAM-labeled aptamers on ice for 30 min in the dark. Figure 4 showed the confocal fluorescence image results. Cells treated with aptamers were brightly fluorescent (Figure 4B and 4C), while fluoresced weakly when treated with unselected library (Figure 4A). In contrast, there was no observable fluorescence signal from control SVGp12 cells treated with aptamers or unselected library (Figure 4D–E), confirming the binding specificity of the selected aptamers.

**Figure 5:**
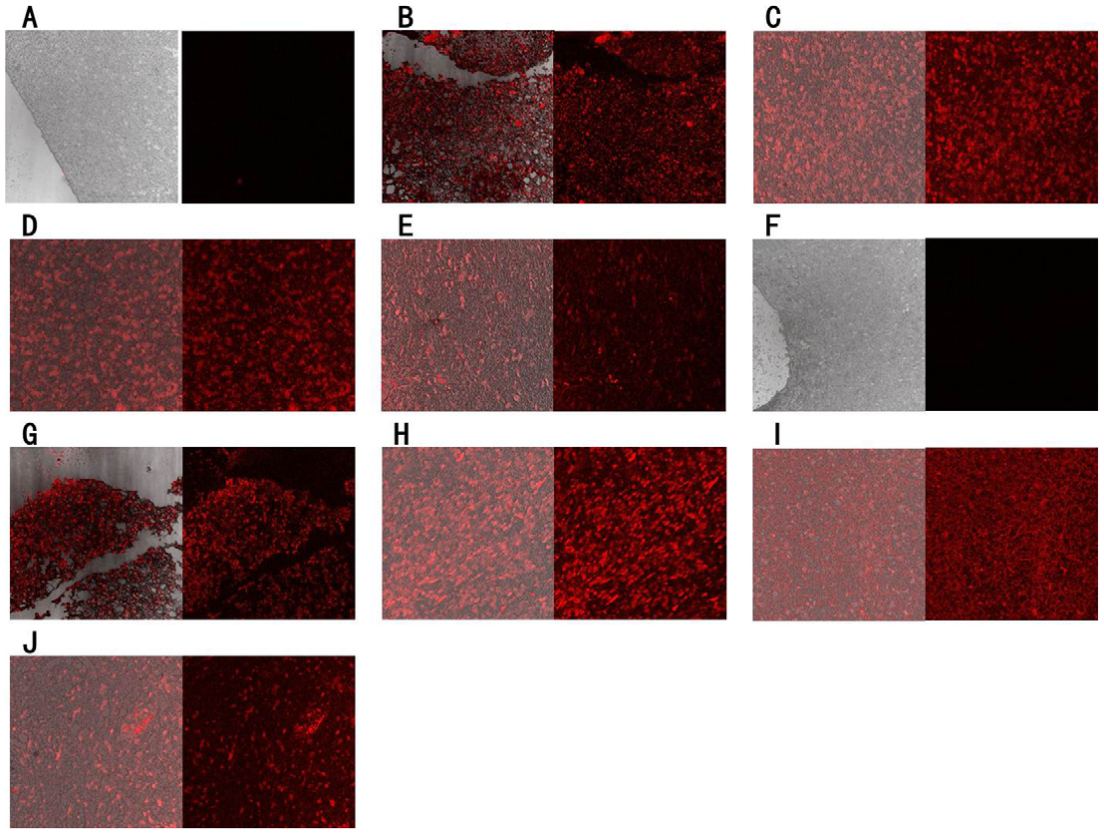
Using the selected aptamers to recognize FFPE normal or glioma tissue sections. FFPE tissue sections were incubated with cy5-labeled aptamers. A =  Normal brain tissue with GBM128; B =  Glioblastoma tissue with GBM128; C =  Anaplastic oligodendroglima with GBM128; D =  Oligoastrocytoma with GBM128; E =  Pilocytic astrocytoma with GBM128; F =  Normal brain tissue with GBM131; G =  Glioblastoma tissue with GBM131; H =  Anaplastic oligodendroglima with GBM131; I =  Oligoastrocytoma with GBM131; J =  Pilocytic astrocytoma with GBM131. The final concentration of Cy5-labeled aptamers was 250 nM.

The results we got so far have clearly proven that the selected two aptamers did show good binding ability towards human glioblastoma cell line U118-MG but not bind to the human astroglial cell line SVGp12. However, whether these two aptamers could recognize glioblastoma cells in clinical tissue samples still needed to be validated. Therefore, formalin-fixed paraffin-embedded (FFPE) slides of normal human astroglial tissues, glioblastoma tissues and other glioma tissues were stained with the Cy5-labeled aptamers. The results of fluorescence imaging were shown in Figure 5. These two aptamers had no binding capability to the normal astroglial tissues (Figure 5A and 5F) but showed significantly strong fluorescence intensity with glioblastoma tissue section (Figure 5B and 5G) and other glioma tissue sections (Figure 5C–E and 5H–J). Several other human cancer tissues, including breast cancer, renal cell carcinoma, medulloblastoma, hepatocellular carcinoma, small cell lung cancer, cervical squamous cell carcinoma, pituitary adenomas, acoustic neuroma, ependymoma and craniopharyngioma, were further tested. Aptamer GBM128 was found to bind weakly to hepatocellular carcinoma tissue (Figure S4D) and ependymoma tissue (Figure S4I) but not to other cancer tissues tested (Figure S4A–C, E–H, J). Aptamer GBM131 was found to bind weakly to medulloblastoma tissue (Figure S5C) and pituitary adenomas tissue (Figure S5G) but not to other cancer tissues tested (Figure S5A, B, D–F, H–J). On the contrary, these two aptamers can bind to globlastoma tissues strongly (Figure S4K and S5K). These results strongly suggested that these two aptamers, GBM128 and GBM131, have the potential to clinically differentiate the glioma tissue from normal astroglial tissue and other human cancer tissues.

In conclusion, cell-SELEX was performed to identify single-stranded DNA ligands against cultured human glioblastoma cell line U118-MG. Two target-specific aptamers named GBM128 and GBM131 were discovered after 30 rounds selection. These two aptamers have high affinity and specificity against target glioblastoma cells. Both aptamers neither recognize normal astraglial cells, nor do they recognize other normal and cancer cell lines tested. Clinical tissues were also tested and the results showed that these two aptamers can bind to different clinical glioma tissues but not normal brain tissues. More importantly, binding affinity and selectivity of these two aptamers were retained in complicated biological samples. The aptamer sequences reported here could be further used for molecular imaging of glioblastoma, targeted drug therapy, ligand guided surgery and biomarker discovery.

## Materials and Methods

### Cell lines and cell culture

Human glioblastoma cell line U118-MG, human astroglial cell line SVGp12, human breast cancer cell line MCF-7, MDA-MB-231 and human normal breast epithelium cell line MCF-10A were purchased from American Type Culture Collection (ATCC). Human glioblastoma cell lines U251-MG, U87-MG, A172, human lung adenocarcinoma epithelial cell line A549, human cervical cancer cell line HeLa, human liver cancer cell line QGY-7703 and human normal liver cell line QSG-7701 were purchased from the Cell Bank of the Chinese Academy of Sciences (Shanghai, China). Human kidney epithelial cell line HEK-293T/17, human colorectal adenocarcinoma cell line HT-29 and human gastric carcinoma cell line KATO III, all originally from ATCC, were kindly provided by Professor Wei Duan of School of Medicine, Deakin University, Australia. All cell lines mentioned above were cultured at 37°C in a humid atmosphere with 5% CO_2_. The growth medium for U118-MG, U87-MG, U251-MG, A172, HeLa, HEK-293T/17 is composed of DMEM supplemented with 10% fetal bovine serum and penicillin-streptomycin. The growth medium for human astroglial cell line SVGp12 is composed of eagle's MEM supplemented with 10% fetal bovine serum and penicillin-streptomycin. The growth medium for MCF-7, MDA-MB-231, human lung adenocarcinoma epithelial cell line A549, human liver cancer cell line QGY-7703 and human normal liver cell line QSG-7701 cells is composed of RPMI-1640 supplemented with 10% fetal bovine serum and penicillin-streptomycin. The growth medium for MCF-10A contains several additives and is composed of DMEM/F12 supplemented with 5% horse serum, 20 ng/mL epidermal growth factor (EGF), 10 μg/mL insulin, 0.5 μg/ml hydrocortisone, 100 ng/mL cholera toxin and penicillin-streptomycin as described in reference [31]. The growth medium for HT-29 is McCoy's 5a Medium Modified supplemented with 10% fetal bovine serum and penicillin-streptomycin. The growth medium for KATO III is IMDM supplemented with 10% fetal bovine serum and penicillin-streptomycin.

### Buffers

The washing buffer (WB) used contained 4.5 g/L glucose, and 5 mM MgCl_2_ in PBS (pH = 7.4). The binding buffer used during selection was prepared by adding yeast tRNA (0.1 mg/mL) and BSA (1 mg/mL) to washing buffer to reduce background binding.

### Primers and ssDNA library

The sense primer is labeled at the 5′-end with FAM or Cy5 to monitor the progress of selection by flow cytometry. The antisense primer is labeled at the 5′-end with biotin to separate the sense and antisense strands after PCR by streptavidin-biotin interaction and followed by alkaline denaturation (0.2 M NaOH). The sense primer 5′-GAA TTC AGT CGG ACA GCG-3′ and antisense primer 5′-GGG AGA CGA TAT TCG TCC ATC-3′ adopted from reference [32] were synthesized using standard phosphoramidite chemistry and purified by RP-HPLC to remove the truncated DNA fragments produced in the chemical synthesis and finally desalted using NAP-5 desalting columns (GE healthcare). The initial library (5′-GAA TTC AGT CGG ACA GCG-N45-GAT GGA CGA ATA TCG TCT CCC-3′, 84 mer) consisting of a pool of oligonucleotides with a continuous stretch of 45 randomized nucleotides flanked on both sides by fixed sequences used for the hybridization with PCR primers during subsequent rounds of amplification were synthesized by Sangon Biotech (Shanghai) Co., Ltd.

### Cell-SELEX procedure

For the first round selection, 5 nmol initial ssDNA library was mixed thoroughly with 500 μL binding buffer, then the mixture was heated at 95°C for 5 min and cooled on ice for 10 min immediately. At least 106 U118-MG cells (a 100-mm ×20-mm culture dish) were incubated with the initial ssDNA library on ice for 1 h on a rotary shaker. After incubation, cells containing the binding sequences were collected and centrifuged at 1000 rpm for 3 min at 4°C. The recovery of the sequences binding to U118-MG cells were achieved by heating the cell-DNA complex at 95°C for 5 min in 500 μL water and then amplified by PCR. The thermal cycling conditions were as follows: 94°C for 3 min (initial denaturation), 10 cycles of 94°C for 30 s, 49.5°C for 30 s, and 72°C for 30 s, followed by a single final extension at 72°C for 5 min. To determine the optimum number of cycles for preparative PCR, PCR amplification was performed using the PCR program mentioned above and products at 5, 7, 9, 11, 13 cycles were taken respectively. Samples were loaded in lanes of agarose gel and electrophoresis was carried out at 100 V for 30 min. The gel was stained with ethidium bromide, and the bands were observed under UV light and taken image. The cycle number that yielded bright band without nonspecific amplification was selected. In preparative PCR, the optimum cycle of amplification was applied to produce more PCR products for the preparation of ssDNA required for the next round of selection. Preparation of ssDNA from PCR product was carried out as described below. The antisense strand is labeled at the 5′-end with biotin, helping to separate the sense and antisense strands after PCR by streptavidin-biotin interaction, followed by alkaline denatura­tion using 500 μL of 0.2 M NaOH. Subsequently, the eluate was collected and desalted by a NAP-5 column. The concentration of ssDNA was determined by UV absorbance at 260 nm. Finally, the ssDNA was concentrated using a DNA Speed Vacuum dryer for the next round of selection. From the second round, the negative selection was introduced, after incubated with target cells, the ssDNA were eluted by heating at 95°C in 500 μL binding buffer. Then centrifuged at 10,000 rpm for 5 min and the supernatant containing DNA was incubated with SVGp12 cells (a 100-mm ×20-mm culture dish, about 107–108 cells) for an hour to remove sequences that may bind to the molecules on both U118-MG and SVGp12 cells. For subsequent rounds of selection (29 rounds in this work), the same steps as outlined above was followed, but with the following modifications as selection progresses. The number of target cells was gradually reduced to increase the stringency of the selection. A 100-mm ×20-mm culture dish was used for the first 4 rounds and a 60-mm ×15-mm culture dish for the fifth round and beyond. At the same time, incubation time for the positive incubation was gradually reduced from 1 h to 30 min, while keeping the cell number and incubation time unchanged for the control selection. Moreover, both washing time and washing buffer volume were gradually increased from 1 min to 15 min and 1 mL to 10 mL respectively. 10% of FBS were added to the binding buffer from the fourth round of selection and gradually increased to 20%.

### Binding assays

To monitor the enrichment of pools during the selection or investigate the binding affinity of aptamer candidates, about 3×105 cells were incubated with final concentration of 250 nM enriched pools or aptamer candidates labeled at the 5′-end with FAM in binding buffer containing 10% FBS on ice for 30 min in the dark, the unselected initial library with the same fluorophore was used as control. Subsequently, cells were washed three times with ice cold washing buffer (with 0.1% Na2N3) and collected then centrifuged at 1000 rpm for 3 min at 4°C. Finally, cells were resuspended in 200 μL washing buffer (with 0.1% Na2N3) and subjected to flow cytometry. The fluorescence intensity of FAM labeled pools was determined in triplicate with flow cytometer (Becton Dickinson Immunocytometry Systems) by counting 10000 events and analyzed using WinMDI 2.9 software (Scripps, La Jolla, CA).

### Cloning and sequencing of enriched pools

When pools with apparent increased fluorescence compared with the unselected initial library, selection was completed. PCR products with the cleanest band were selected for cloning. The positive clones were sequenced (Genomics Institute, Shenzhen) to identify individual aptamer candidates. The resulting 246 sequences were subjected to multiple sequence alignment analysis using ClustalX2.0.3 software [33] to discover highly conserved motifs in groups of selected DNA sequences. The discovered consensus sequences with high repeats among selected pools were then chemically synthesized on DNA synthesizer (Polygen, Germany) for further study.

### Determination of dissociation constant (K_d_) value for aptamers

To determine the binding affinities of aptamers, U118-MG cells (3×10^5^) were incubated with various concentrations of FAM-labeled aptamers in binding buffer on ice for 30 min in the dark. Cells were then washed three times with washing buffer (with 0.1% Na2N3), then resuspended in 200 μL binding buffer (with 0.1% Na2N3) and subjected to flow cytometry analysis. The mean fluorescence intensity of U118-MG cell-aptamer complex was used to evaluate binding affinity by subtracting the mean fluorescence intensity of non-specific binding produced by unselected initial library. Using SigmaPlot software (Jandel Scientific), K_d_ of the aptamer-cell interaction was obtained by fitting the dependence of fluorescence intensity of specific binding on the concentration of aptamer which determined in triplicate to the one-site saturation equation Y = B_max_ X/(K_d_+X).

### Investigation of binding specificity of aptamers by flow cytometry

To investigate the specificity of aptamers for molecular recognition of U118-MG cells, control cell line SVGp12 and other related gliblastoma cell lines such as U87-MG, U251-MG, A172, human breast cancer cell lines MCF-7 and MDA-MB-231, huaman normal breast epithelium cell line MCF-10A, human lung adenocarcinoma epithelial cell line A549, human liver cancer cell line QGY-7703, human normal liver cell line QSG-7701, human cervical cancer cell line HeLa, human kidney epithelial cell line HEK 293T/17, human colorectal adenocarcinoma cell line HT-29, and gastric carcinoma cell line KATO III cells were also applied for binding specificity assays with aptamer candidates and unselected initial library by flow cytometry respectively.

### Investigation of binding specificity of aptamers by laser confocal fluorescence microscopy

To further characterize the specificity of aptamers, fluorescence images of U118-MG and SVGp12 cells bound with aptamers or unselected initial library labeled with FAM were taken under laser confocal fluorescence microscopy (Leica, Germany). Cells cultured into monolayers with about 60% confluence were used. First of all, U118-MG and SVGp12 cells were exposed to 250 nM of FAM-labeled aptamers or unselected initial library in binding buffer on ice for 30 min in the dark. Next, supernatant was removed and cells were washed three times with ice-cold washing buffer (with 0.1% Na_2_N_3_). Finally, fluorescence images of the cell were taken.

### Staining of human tumor tissue sections using selected aptamers

The formalin-fixed paraffin-embedded (FFPE) tissue slides (5 μm thickness) were supplied by the Department of Pathology of the First Affiliated Hospital of Fujian Medical University (Fuzhou, China). The pre-treatment of FFPE tissue slides was performed as described in references [34], [35]. Briefly, all the tissue sections were deparaffinized in xylene (5 min×3), then rehydrated through a degraded ethanol series (100%, 95% and 70%) for 20 s respectively. After washing with TE buffer (pH = 8.0), the hydrated tissue sections were heated in this buffer at 95°C for 20 min to retrieve antigens and cooled slowly in this buffer. The tissue sections were then incubated with 200 mL binding buffer containing 10% FBS of 250 nM Cy5-labeled aptamers for 30 min on ice in the dark. Then these tissue sections were washed three times with binding buffer, dehydrated and sealed. Finally, the stained sections were imaged by laser confocal fluorescence microscopy (Leica, Germany). Cy5 was excited by a 633 nm argon ion laser.

## Supporting Information

Figure S1 **The binding affinity of aptamers GBM128 and GBM131 with U118 MG and SVGp12 cells.** A: Aptamer GBM128 and GBM131 could bind with U118-MG cells very well. B: Control SVGp12 cells showed no binding with aptamers GBM128 and GBM131. Final concentration of FAM-labeled aptamers was 250 nM in binding buffer.
(TIF)

Figure S2 **Effect of temperature on the binding affinity of aptamers GBM128 and GBM131.** Aptamers GBM128 and GBM131 can bind very well to U118-MG cells at 4°C (A and B). At 37°C, aptamer GBM128 lost its binding ability to U118-MG cells (C), however, aptamer GBM131 can bind to U118-MG cells still though its fluoresce intensity was smaller than at 4°C (D). The final concentration of FAM-labeled aptamers is 250 nM.
(TIF)

Figure S3 **The binding affinity of aptamers GBM128 and GBM131 with U118 MG in complete cell growth medium.** Aptamer GBM128 and GBM131 could still bind with U118-MG cells very well in growth medium.
(TIF)

Figure S4 **Using aptamer GBM128 to recognize different FFPE caner tissues.** Different FFPE tissue sections were incubated with cy5-labeled aptamer GBM128. A =  Brest cancer tissue; B =  Renal cell carcinoma tissue; C =  Medulloblastoma tissue; D =  Hepatocellular carcinoma tissue; E =  Small cell lung cancer tissue; F =  Cervical squamous cell carcinoma tissue; G =  Pituitary adenomas tissue; H =  Acoustic neuroma tissue; I =  Ependymoma tissue; J =  Craniopharyngioma tissue; K =  Glioblastoma tissue. The final concentration of Cy5-labeled aptamers was 250 nM.
(TIF)

Figure S5 **Using aptamer GBM131 to recognize different FFPE caner tissues.** Different FFPE tissue sections were incubated with cy5-labeled aptamer GBM131. A =  Brest cancer tissue; B =  Renal cell carcinoma tissue 8; C =  Medulloblastoma tissue; D =  Hepatocellular carcinoma tissue; E =  Small cell lung cancer tissue; F =  Cervical squamous cell carcinoma tissue; G =  Pituitary adenomas tissue; H =  Acoustic neuroma tissue; I =  Ependymoma tissue; J =  Craniopharyngioma tissue; K =  Glioblastoma tissue. The final concentration of Cy5-labeled aptamers was 250 nM.
(TIF)

Table S1 **Specificity of aptamer candidates.** Selectivity study of selected aptamers to different cell lines including glioblastoma cell lines (U118-MG, U87-MG, U251, A172), astroglial cell line (SVGp12), normal breast epithelium cell line (MCF-10A), breast cancer cell lines (MCF-7, MDA-MB-231), lung cancer cell line (A549), normal liver cell line (QSG-7701), liver cancer cell line (QGY-7703), human cervical cancer cell line HeLa, human kidney epithelial cell line HEK-293T/17, human colorectal adenocarcinoma cell line HT-29, human gastric carcinoma cell line KATO III. Aptamers GBM128 and GBM131 showed high specificity. + for binding; − for no binding; ND for no detection.
(DOC)
