# Supplementary figures and images for: Selection of DNA Aptamers against Glioblastoma Cells with High Affinity and Specificity
Source: PLoS One. 2012 Oct 2;7(10):e42731. doi: 10.1371/journal.pone.0042731 (PMC3462804; doi:10.1371/journal.pone.0042731)

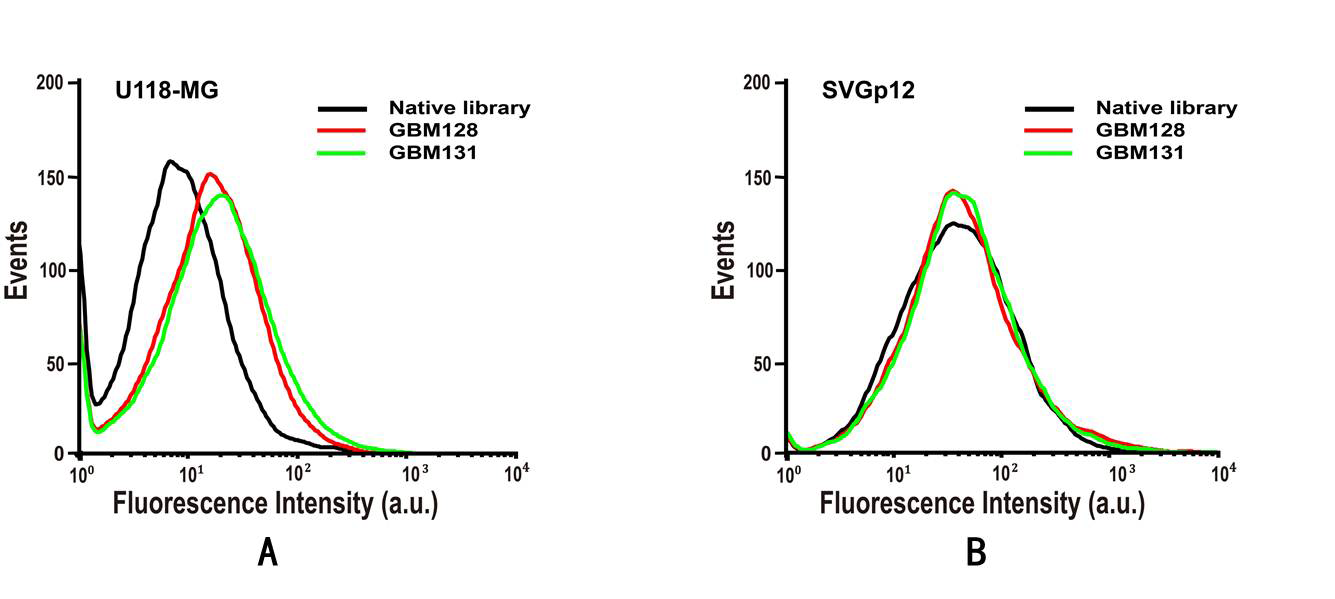

Supplement: Figure S1 — The binding affinity of aptamers GBM128 and GBM131 with U118 MG and SVGp12 cells. A: Aptamer GBM128 and GBM131 could bind with U118-MG cells very well. B: Control SVGp12 cells showed no binding with aptamers GBM128 and GBM131. Final concentration of FAM-labeled aptamers was 250 nM in binding buffer. (TIF) [file pone.0042731.s001.tif]

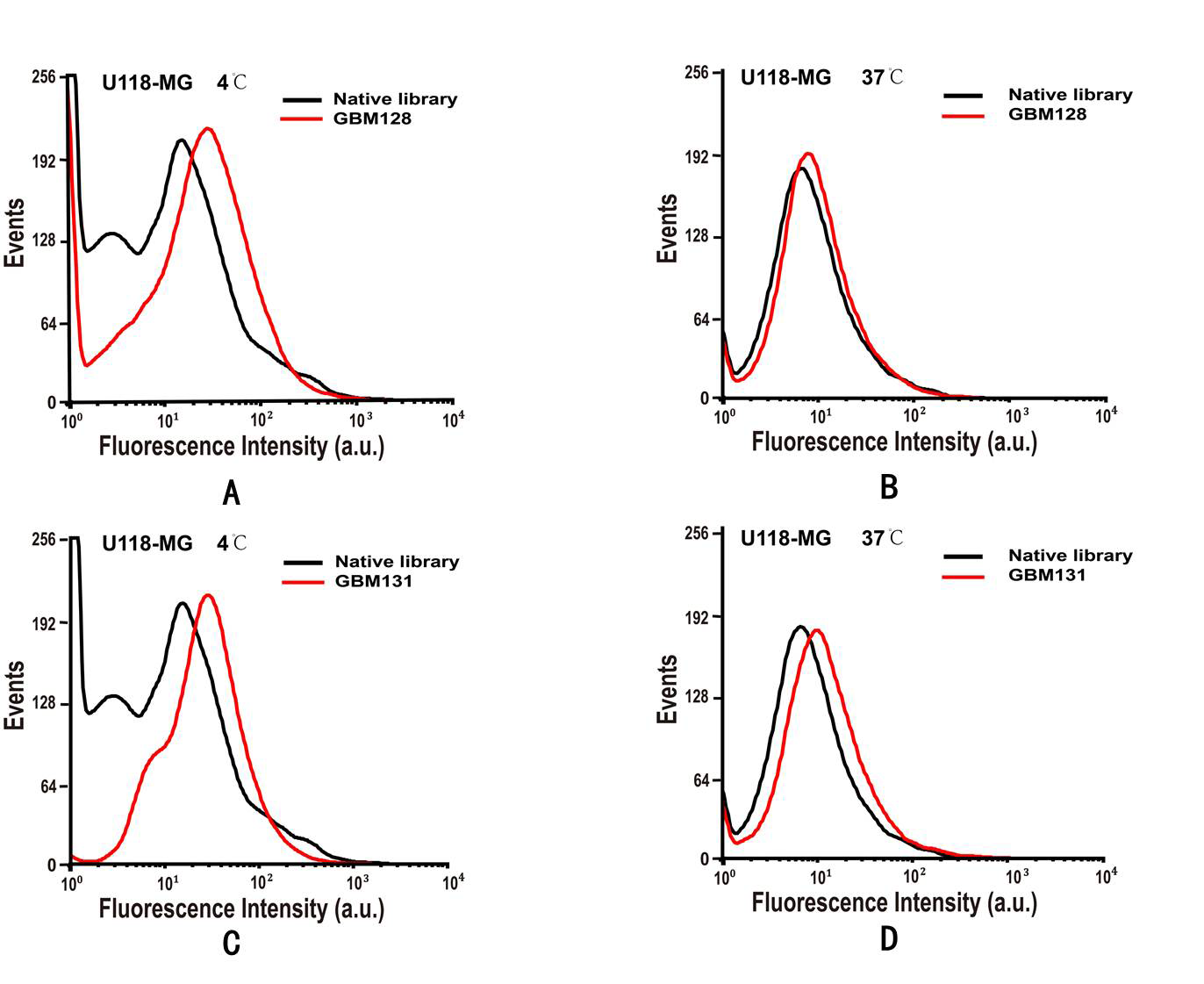

Supplement: Figure S2 — Effect of temperature on the binding affinity of aptamers GBM128 and GBM131. Aptamers GBM128 and GBM131 can bind very well to U118-MG cells at 4°C (A and B). At 37°C, aptamer GBM128 lost its binding ability to U118-MG cells (C), however, aptamer GBM131 can bind to U118-MG cells still though its fluoresce intensity was smaller than at 4°C (D). The final concentration of FAM-labeled aptamers is 250 nM. (TIF) [file pone.0042731.s002.tif]

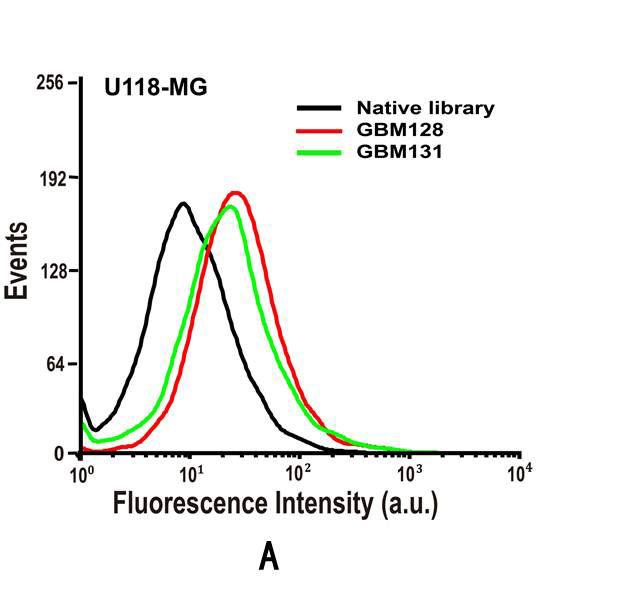

Supplement: Figure S3 — The binding affinity of aptamers GBM128 and GBM131 with U118 MG in complete cell growth medium. Aptamer GBM128 and GBM131 could still bind with U118-MG cells very well in growth medium. (TIF) [file pone.0042731.s003.tif]

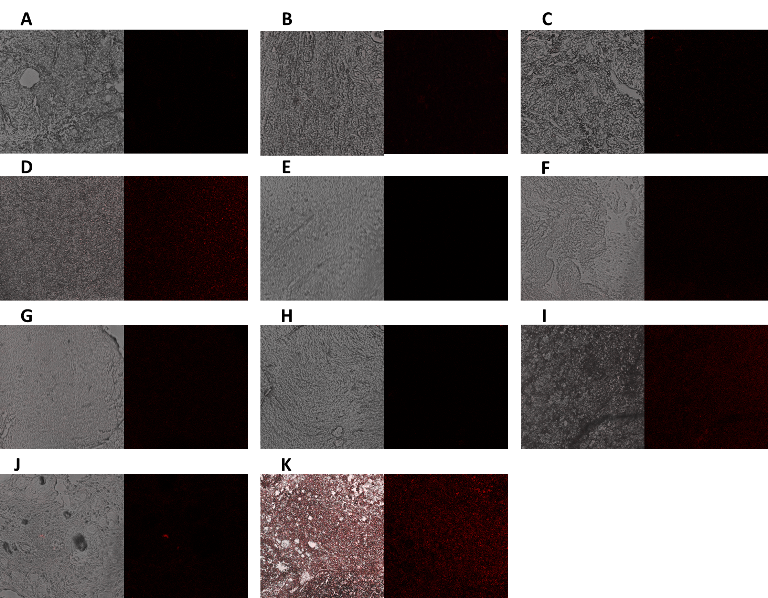

Supplement: Figure S4 — Using aptamer GBM128 to recognize different FFPE caner tissues. Different FFPE tissue sections were incubated with cy5-labeled aptamer GBM128. A = Brest cancer tissue; B = Renal cell carcinoma tissue; C = Medulloblastoma tissue; D = Hepatocellular carcinoma tissue; E = Small cell lung cancer tissue; F = Cervical squamous cell carcinoma tissue; G = Pituitary adenomas tissue; H = Acoustic neuroma tissue; I = Ependymoma tissue; J = Craniopharyngioma tissue; K = Glioblastoma tissue. The final concentration of Cy5-labeled aptamers was 250 nM. (TIF) [file pone.0042731.s004.tif]

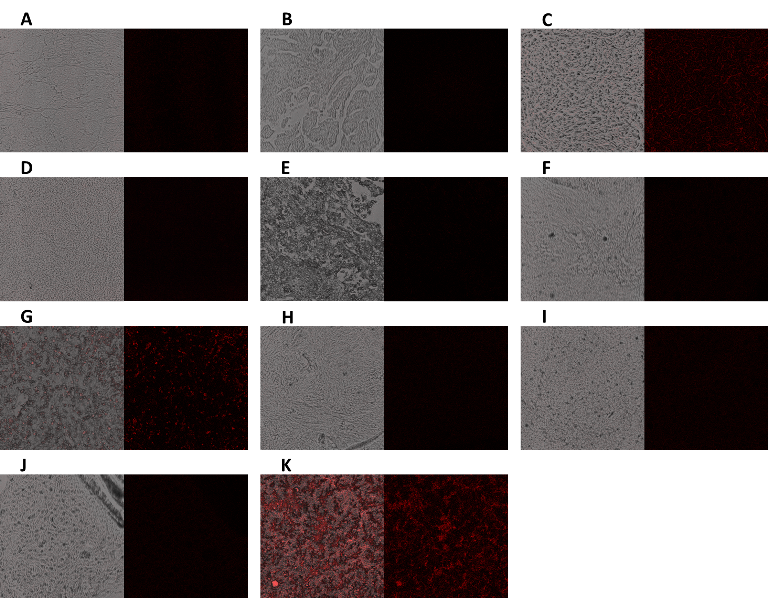

Supplement: Figure S5 — Using aptamer GBM131 to recognize different FFPE caner tissues. Different FFPE tissue sections were incubated with cy5-labeled aptamer GBM131. A = Brest cancer tissue; B = Renal cell carcinoma tissue 8; C = Medulloblastoma tissue; D = Hepatocellular carcinoma tissue; E = Small cell lung cancer tissue; F = Cervical squamous cell carcinoma tissue; G = Pituitary adenomas tissue; H = Acoustic neuroma tissue; I = Ependymoma tissue; J = Craniopharyngioma tissue; K = Glioblastoma tissue. The final concentration of Cy5-labeled aptamers was 250 nM. (TIF) [file pone.0042731.s005.tif]
