# Supplementary material for: Selection of DNA Aptamers against Glioblastoma Cells with High Affinity and Specificity
Source: PLoS One. 2012 Oct 2;7(10):e42731. doi: 10.1371/journal.pone.0042731 (PMC3462804; doi:10.1371/journal.pone.0042731)
Supplement: Table S1 — Specificity of aptamer candidates. Selectivity study of selected aptamers to different cell lines including glioblastoma cell lines (U118-MG, U87-MG, U251, A172), astroglial cell line (SVGp12), normal breast epithelium cell line (MCF-10A), breast cancer cell lines (MCF-7, MDA-MB-231), lung cancer cell line (A549), normal liver cell line (QSG-7701), liver cancer cell line (QGY-7703), human cervical cancer cell line HeLa, human kidney epithelial cell line HEK-293T/17, human colorectal adenocarcinoma cell line HT-29, human gastric carcinoma cell line KATO III. Aptamers GBM128 and GBM131 showed high specificity. + for binding; − for no binding; ND for no detection. (DOC) [file pone.0042731.s006.doc]

|  | ***GBM1*** | ***GBM7*** | ***GBM10*** | ***GBM11*** | ***GBM17*** | ***GBM24*** | ***GBM34*** | ***GBM128*** | ***GBM131*** |
| --- | --- | --- | --- | --- | --- | --- | --- | --- | --- |
| **U118-MG** | **＋** | **＋** | **＋** | **＋** | **＋** | **＋** | **＋** | **＋** | **＋** |
| **SVGp12** | **＋** | **－** | **＋** | **＋** | **＋** | **＋** | **＋** | **－** | **－** |
| **A172** | **－** | **﹣** | **＋** | **＋** | **＋** | **－** | **＋** | **－** | **－** |
| **U87-MG** | **＋** | **＋** | **＋** | **＋** | **＋** | **＋** | **＋** | **－** | **＋** |
| **U251-MG** | **＋** | **－** | **＋** | **＋** | **＋** | **－** | **＋** | **－** | **－** |
| **MCF-10A** | **＋** | **＋** | **＋** | **＋** | **＋** | **＋** | **＋** | **－** | **－** |
| **MCF-7** | **＋** | **－** | **＋** | **＋** | **＋** | **＋** | **＋** | **－** | **－** |
| **MDA-MB-231** | **＋** | **＋** | **＋** | **＋** | **＋** | **－** | **＋** | **－** | **－** |
| **A549** | **＋** | **－** | **＋** | **＋** | **＋** | **－** | **＋** | **－** | **－** |
| **QSG-7701** | **＋** | **＋** | **＋** | **＋** | **＋** | **－** | **＋** | **－** | **－** |
| **QGY-7703** | **－** | **＋** | **＋** | **＋** | **＋** | **－** | **＋** | **－** | **－** |
| **HeLa** | **＋** | **－** | **＋** | **＋** | **＋** | **－** | **＋** | **－** | **－** |
| **HEK-293T/17** | **ND** | **ND** | **ND** | **ND** | **ND** | **ND** | **ND** | **－** | **－** |
| **HT-29** | **ND** | **ND** | **ND** | **ND** | **ND** | **ND** | **ND** | **－** | **－** |
| **KATO Ⅲ** | **ND** | **ND** | **ND** | **ND** | **ND** | **ND** | **ND** | **－** | **－** |
